# Hydrophobisation of Silica Nanoparticles Using Lauroyl Ethyl Arginate and Chitosan Mixtures to Induce the Foaming Process

**DOI:** 10.3390/polym14194076

**Published:** 2022-09-28

**Authors:** Marcel Krzan, Ewelina Jarek, Hristina Petkova, Eva Santini, Lilianna Szyk-Warszynska, Francesca Ravera, Libero Liggieri, Elena Mileva, Piotr Warszynski

**Affiliations:** 1 Jerzy Haber Institute of Catalysis and Surface Chemistry, Polish Academy of Sciences, ul. Niezapominajek 8, 30-239 Krakow, Poland; 2 Institute of Physical Chemistry, Bulgarian Academy of Sciences, Acad. G. Bonchev Str., bl. 11, 1113 Sofia, Bulgaria; 3 Institute of Condensed Matter Chemistry and Technologies for Energy, Consiglio Nazionale delle Ricerche, Via Marini 6, 16149 Genoa, Italy

**Keywords:** chitosan, biopolymers, lauroyl ethyl arginate (LAE), cationic surfactants, particle hydrophobisation, foaming

## Abstract

We studied silica suspensions with chitosan and biodegradable synthetic surfactant lauroyl ethyl arginate (LAE). Hydrophilic and negatively charged silica nanoparticles were neutralised due to the coating with chitosan. That presence of LAE led to the partial hydrophobisation of their surface, which favoured their attachment to the surface of a thin foam film. It was found that the presence of small and medium-sized (6–9 nm) hydrophobic particles in the interfacial layer of lamella foam film inhibited the coalescence and coarsening processes, which prolonged the life of the foam. Furthermore, hydrophobising of 30 nm particles allowed the formation of large aggregates precipitating from the mixture under steady-state conditions. These aggregates, however, under the conditions of the dynamic froth flotation process in the foam column, were floated into the foam layer. As a result, they were trapped in the foam film and Plateau borders, effectively preventing liquid leakage out of the foam. These results demonstrate the efficiency of using chitosan-LAE mixtures to remove silica nanoparticles from aqueous phase by foaming and flotation.

## 1. Introduction

In recent years, the presence of ultrafine nanoparticles in waters due to human activities has increased significantly. Nanoparticles and materials enriched with nanoparticles are widely used in industry, and waste from producing and using such materials ends up in the aquatic environment. As a result, organic or synthetic nanoparticles contaminating drinking water sources and groundwater threaten human health, life, and aquatic fauna [1].

It is commonly believed that due to the original hydrophobic properties of nanoplastics, similar contaminants can be easily removed by foam flotation. For example, polystyrene, polyurethane and other components of microplastics exhibit significant hydrophobic properties [2,3]. However, recent studies have shown that similar microparticles easily interact in an aqueous environment with hydrophilic and oppositely charged chemicals (surfactants, antibiotics or fertiliser ingredients), thereby changing their surface properties from hydrophobic to hydrophilic [4]. The consequence is the inability to remove them in the classical froth flotation process. As a result, nanoplastics remain in the waters and cause increasing environmental damage.

These particles are too small to sediment and display hydrophilic properties, effectively preventing them from aggregation or from being attached to any surface [5]. Moreover, solid particles in aqueous suspension are surrounded by an electrical double layer (EDL). This creates an additional energy barrier that hinders the coalescence and interaction of particles with each other and with interfaces. Therefore, we must change their surface charge and hydrophilic properties to remove them from the solution. All this must be achieved using the most environmentally friendly ways possible. This paper presents the possibilities of eliminating such pollutants by froth flotation using a mixture of LAE and chitosan as hydrophobisation and surface charge neutralisation agents. We assume that due to the flotation process, the particles will be transferred into a stable foam fraction.

Flotation is a separation technique based on capturing hydrophobic particles by bubbles and their collection in a froth layer [6,7]. This process was developed to separate hydrophobic particles of a useful mineral from hydrophilic particles of sand and other non-useful minerals. There are usually two kinds of chemical compounds involved in this process, the frother and the collector. A frother (surface active agent) reduces the surface tension of the liquid-gas interface to enable froth formation, hinder coalescence from stabilising bubble size and facilitate hydrophobic particle adhesion to air bubbles. At the same time, a collector is a type of organic compound that selectively attaches to the surface of the minerals and adds water repelling nature to the particles, which is a critical factor for the adhesion of mineral particles to the air bubble.

Flotation has already been used to remove pollutants from water. However, every literature report describes only the separation of hydrophobic contaminants from other hydrophilic clays or minerals [8,9]. We believe it will be possible to modify the classical flotation process and apply it to negatively charged and hydrophilic nanoparticles using proper chemicals for particle surface charge neutralisation and hydrophobisation.

Aqueous foams are complex systems with an internal cellular structure consisting of polydisperse gas bubbles separated by thin liquid films and Plateau borders [10,11,12,13,14,15,16,17,18,19]. Foam evolution and its transient stability are functions of drainage, gas diffusion and the rupture of liquid films between air bubbles (coalescence). The primary process in wet foams is free drainage, governed by gravitational acceleration and a viscous force acting between the adjacent bubbles [20,21,22]. By drainage, we refer to the irreversible flow of liquid through the foam, which leads to liquid accumulation at the foam’s bottom. Due to the drainage, the total (or overall) liquid content decreases within the foam. As a result, on the top of the foam column, dry foams with polyhedral bubbles with thin edges are formed, while the bubbles in the bottom part of the foams are still spherical. When the liquid films between the bubbles are very thin, they eventually break, and the bubble coalescence or foam collapse can happen. Coarsening (gas diffusion) involves the transport of gas between bubbles of different sizes, leading to the growth of the larger bubbles. It occurs when suitable stabilising agents are absent.

Obtaining a stable particle-laden foam, i.e., resistant to mechanical disturbances, as a result of the flotation process requires the fulfilment of many physical, chemical and mechanical conditions [5,23]. The problem of particle attachment to the interface is more difficult to understand than in the case of surfactant adsorption. This is because particles mostly exhibit neither surface activity nor foamability properties. Moreover, only the particles with appropriate sizes, shapes, hydrophobic properties and surface potentials can be attached to the interface [5,23]. As a result, the attachment of particles to the interfacial layer is not spontaneous, like the adsorption of surfactant molecules. Therefore, generating aqueous foams stabilised solely by solid particles requires strong mechanical methods, such as mechanical homogenisation, shaking, turbulent mixing or whipping [24,25,26,27,28,29,30,31,32].

On the other hand, foams stabilised with micro- and nanoparticles have several interesting properties from the point of view of science and technology. They have better stability and noticeable yield stress [33,34]. Particles can also control the foam lift-time due to their various specific properties. As an example, iron or iron oxide particles can be mentioned, which at the beginning can work as foam stabilisers, but later destroy the foam via the application of an external magnetic field [34,35,36,37]. Carbon particles can also initially stabilise foam and later destroy it with ultraviolet radiation [38]. The viscoelastic response of particle-stabilised foams is different from that of classic foams [33,39], which typically show viscous behaviour without an elastic modulus or yield stress. The presence of yield stress in particle-laden foams is connected with the irreversible attachment of the particles to the foam film surfaces. The particles are immobilised in some places of the foam films and engage in mutual interaction to generate some rigid structure. External stresses can either shift the whole foam to a new state or destroy the particles’ mutual interactions and contacts with the foam films, leading to a collapse of the foam. The variations in the viscoelasticity with the solid volume fraction were in qualitative agreement with that predicted by an effective medium rigidity particle concentration model. The network of uniform and equal springs relevant for classical foams was transformed into another structure consisting of very strong springs generated by rigid bonds between particles [33,39]. It was also found that there is a maximum concentration of particles above which the viscoelastic properties are no longer improved. That maximum concentration increases with the particles’ size. It was concluded that the studied particle-laden foams behaved like a superelastic material [33].

Hydrophilic particles do not attach to foam film surfaces but remain dispersed in the bulk of the foam films. They can only, at a certain concentration, diminish the liquid flow during drainage due to viscosity effects [25]. Therefore, in the case of hydrophilic particles, it is necessary to carry out hydrophobisation of the particles in solution using an appropriate surfactant. This compound then acts not only as a frother but also as a hydrophobising agent. The partial hydrophobisation of metalloid particles by short chain carboxylic acids, alkyl gallates or alkylamines as amphiphiles was presented in a series of papers [40,41,42,43,44,45]. It created stable foams that did not collapse even after the complete drainage. The spontaneous adsorption of the surfactants on the surface of the particles was caused by specific electric interactions (carboxylates and amines) and ligand-exchange reactions. It has been shown that this approach can be used to modify colloidal hydrophilic oxide particles with different surface chemistry. The functional group of the surfactant was adapted to the surface of the particles to be selected as the foam stabiliser. It has also been shown that a higher degree of surfactant adsorption on the particle surface leads to more stable foams [40,41,42,43,44,45].

Partially hydrophobic particles (depending on their size, shape, and concentration) can aggregate and accumulate in the Plateau borders and slow down the liquid drainage [26]. Such particles can also form a network structure (some kind of weak gel), which permanently stabilises foam fraction. Superstable particle-laden wet foam can be generated only with particles of the proper size and degree of hydrophobisation. This is connected with the irreversible attachment of the particles to the interfacial layer. The energy of the particle detachment can be as high as several thousand kT, which means the particles’ attachment energy is a thousand times higher than the adsorption energy of a surfactant molecule [27,28].

A similar approach can be used for inorganic and organic particles. Cellulose nanoparticles covered by oppositely charged surfactants can attach to the gas/solution interface, lowering surface tension and modifying the dilational surface viscoelasticity [46,47]. They reduce drainage and prevent coalescence. Large cellulose nanocrystal aggregates significantly affect foam stability, as they accumulate in the Plateau borders and stop liquid drainage [46,47].

We decided to use Levasil^®^ silica nanoparticles as a model of hydrophilic and negative charged particles. Levasil^®^ is an alkaline, aqueous dispersion of colloidal silica. The silica dispersion is stabilised ammonia, and the amorphous silica particles carry a negative surface charge. The silica particles are well dispersed, have a slightly rough, spherical shape and a narrow particle size distribution. The physical appearance of the dispersion is a translucent liquid, slightly more viscous than water. Levasil^®^ is used in paints, floor wax and polishes and printing inks.

We used the natural biopolymer and polycation chitosan as a surface charge neutralisation agent for silica particles. It has been proven to neutralise negatively charged solid surfaces with chitosan without adding cross-linkers or activating agents [48,49].

Chitosan is obtained from the hard outer skeleton of shellfish, including crab, lobster, and shrimp. Chitosan (b-(1-4) linked 2-amino-2-deoxy-D-glucose) is a biocompatible, antibacterial, antifungal and environmentally friendly polysaccharide [50,51,52,53,54,55,56,57,58,59]. It is found in the cell walls of some fungi, but for commercial use, it is produced from chitin by partial alkaline deacetylation of chitin. From the physicochemical point of view, chitosan should be considered as a polyelectrolyte, polycation under acidic conditions [51,52,53,58].

We used a cationic surfactant, lauroyl ethyl arginate (LAE) [46,47,60,61], as the hydrophobising agent and main surface-active component. An interaction between the hydrophilic glass and cationic surfactant was already observed in [48,62,63,64,65]. The LAE was synthesised by esterifying arginine with ethanol, followed by a reaction of the ester with lauroyl chloride [46,47,60,61,66,67,68,69,70,71,72,73]. The resultant ethyl lauroyl arginate was recovered as a hydrochloride salt. LAE has a wide spectrum of activity against Gram-positive and negative bacteria, yeasts and moulds [68,69,70,71,72,73]. The complex toxicology tests [69] proved the safety of LAE as a food additive. When stored, LAE decomposes by hydrolysis into the surface-active components Nα-lauroyl–l-arginine (LAS) or dodecanoic (lauric) acid. LAE is metabolised in plasma by hydrolysis of the ethyl ester and lauroyl amide to ethanol, lauric acid and arginine. Arginine is further metabolised to ornithine and urea. LAE is already used as a preservative (E-243) in most heat-treated meat products or as a multi-functional component in cosmetics and toiletry formulations (an anti-static agent and/or surfactant with antimicrobial properties). It has been approved and generally recognised as safe (GRAS) for some food and biomedical applications by the USA Food and Drug Administration (FDA) and the European Food Safety Agency (EFSA) [71,72]. LAE interacts with biopolymers due to electrostatic interactions leading to complex formation [72,73].

In this work, we studied the surface-active and foaming properties of mixtures of LAE and chitosan. We also demonstrated the mutual interactions between Levasil^®^’s particles and chitosan, acetic acid and lauroyl ethyl arginate, examining changes in the zeta potential, bulk viscosity and size of particle aggregates in mixtures. Then, we studied the effect of adding Levasil^®^ of various sizes (6, 9 and 30 nm) into mixtures of LAE and chitosan on their surface activity, foamability and foam stability. Finally, we attempted to correlate surface tension and dilational viscoelastic properties of dispersions with their foamability and foam stability.

Our findings can contribute to developing a safe wastewater treatment method for particles with similar properties. Furthermore, these results can also be used to create various particle-stabilised foams with different cosmetic and medical properties.

## 2. Materials and Methods

### 2.1. Materials

Chitosan (cat. no. 448877)—medium molecular-weight (190–310 kDa) chitosan powders purchased from Sigma–Aldrich (Saint Louis, MO, USA) with a deacetylation degree of about 75–85% were used as received. The chitosan solution was prepared for each experiment by dissolving chitosan powders in 1% wt. acetic acid. The solution was continuously stirred with a sterile magnetic bar for a few hours. The undissolved impurities were removed through filtration.

Acetic acid (cat. no. 568760114), necessary for the chitosan dissolution, was purchased from Avantor Performance Materials Poland S.A. (Gliwice, Poland) with a purity of about 99.5–99.9% CZDA. The compound was used as received.

The Mirenat-M is a commercial name for a cationic surfactant composition of 85% lauroyl ethyl arginate (LAE), produced by Vedeqsa Grupo LAMIRSA (Terrassa, Barcelona, Spain). The active ingredient is the hydrochloride salt of ethyl-Nα-lauroyl-L-arginate (ethyl-Nα-dodecanoyl-L-arginate·HCl, CAS number 60372-77-2), and its molecular weight is 421.02. Mirenat-M was used as received.

The colloidal silica (Levasil^®^ 100/45%, Levasil^®^ 300/30%, Levasil^®^ 500/15%) was purchased from AkzoNobel (Amsterdam, The Netherlands)^®^). The Levasil^®^ is an aqueous, solvent-free colloidal solution of amorphous silicon dioxide with average particle sizes (producer specification): Levasil^®^ 100/45%—30 nm, Levasil^®^ 300/30%—9 nm, Levasil^®^ 500/15%—6 nm. Each studied Levasil^®^ was used as received.

### 2.2. Solution Preparation Methodology

In all our studies, we used mono-component stock solutions of chitosan (1000 ppm) and lauroyl ethyl arginate (500 ppm). Stock solutions were prepared 24 h before experiments, kept in the thermostat at a constant temperature 22 ± 1 °C and used for the experimental mixture preparations just before the measurements.

Chitosan was dissolved in the presence of 1%wt. acetic acid. One gram of chitosan was flooded with 10 mL of pure acetic acid. Next, the solution volume was filled with water to one litre. Finally, the prepared suspension was left on a magnetic stirrer for at least 8 h (overnight) to mix and fully dissolve the chitosan.

Chitosan mixtures with LAE were prepared in the same manner. First, we measured the volume of chitosan solutions necessary to obtain a 100 ppm concentration. Second, in the same way, we added the volume of lauroyl ethyl arginate (also to obtain 100 ppm solutions). Next, the necessary volume of acetic acid was incorporated to obtain the constant acetic acid concentrations of 1% wt. Finally, the experimental mixture was complemented by the required volume of water.

### 2.3. Levasil^®^ Suspension Preparations

For the research, we assigned three types of Levasil^®^ hydrophilic silica nanoparticles with different sizes: Levasil^®^ 100/45%—30 nm, Levasil^®^ 300/30%—9 nm, Levasil^®^ 500/15% —6 nm. To obtain comparable conditions for the experiments, we prepared mixtures always containing the same overall surface area of the silica—90 m^2^ in 100 cm^3^ of the mixture. Table 1 presents the volumes of Levasil^®^ mixtures used in our experiments (per 100 cm^3^ of the total chitosan/Levasil^®^ or chitosan/LAE/Levasil^®^ mixtures).

### 2.4. Foaming and Foam Stability in Foam Column

The foam height evolution measurements to study foam ability and foam stability were performed in the glass column. For the experiments, we used the computer-controlled automatic Anton-Paar (Anton Paar GmbH, Graz, Austria) apparatus containing the foam column with an inner diameter of 45 mm, equipped with a fritted glass (G4) at the bottom. The setup was described in [14,74]. The column was connected to the automatic gas dispenser (syringe pump). Before each experiment, the column was carefully cleaned. The clean and dry column was filled with a constant volume (50 cm^3^) of the studied solutions. The foam was generated in a highly reproducible manner by the automatic dispersion of the air (91 cm^3^). Foam started to form when the number of bubbles arriving (concentrating) at the liquid/gas interface exceeded the number of rupturing bubbles. The dynamic evolution of the foam height and time of the foam fraction existence (period necessary for rupture of all bubbles, i.e., their lifetime) were determined with high resolutions by special optical sensors, even with very short-living foams. To obtain more detailed information about the foam ageing and structure of the foam cells, a digital video camera Toshiba SX900 was used to record the foam evolution.

### 2.5. Foam Film Thickens and Stability

Thin foam film stability experiments were carried out using the microinterferometric method of Scheludko-Exerowa and the Thin-Liquid-Film-Pressure-Balanced Technique (TLF-PBT) [12,22,24]. The study allowed us to determine the thin foam film thickness and stability. Two variants of the experimental cell were used: the so-called Scheludko-Exerowa cell and a porous-plate cell. In the Scheludko-Exerowa cell, the foam film was formed in the middle of a biconcave drop in a glass holder with a radius r = 2 mm. The measuring cell enables foam films’ investigation under constant capillary pressure, determined by the air/solution surface tension (σ) and the glass holder’s diameter. In the Thin-Liquid-Film-Pressure Balanced Technique, the porous plate cell was applied. The technique enabled direct measurement of the disjoining pressure vs. thickness isotherms and allowed one to follow the film’s behaviour within a broad pressure range. In the present experiments, the porous cell used had a horizontally placed capillary to eliminate the impact of the hydrostatic pressure. All film experiments were conducted at room temperature (22 ± 1 °C). Before every measurement, the investigated solution was kept inside the measuring cell for about an hour to reach the required temperature.

### 2.6. Surface Tension and Surface Rheology

The dynamic surface tension measurements were performed with the maximum bubble pressure tensiometer (BPA-1S) manufactured by Sinterface (Sinterface e.K., Berlin, Germany). This experimental methodology was described elsewhere [75,76]. We used the capillary with an inner radius of 0.130 mm for measurements in a dynamic time range between 0.01 and 100 s and standard experimental mode with the maximal bubble lifetime equal to 5 s and the maximal duration of the experiment of 20 min. All experiments were carried out at room temperature of 22 ± 1 °C.

The surface rheological properties were determined using the profile analysis tensiometer Sinterface PAT1M (Sinterface e.K., Berlin, Germany) [77], which is based on the acquisition of the profile of a droplet formed at the tip of a steel capillary. This technique allowed us to perform measurements of the equilibrium and dynamic surface tension following the evolution of the surface tension value with the interface’s ageing, keeping the drop area constant. Moreover, it was possible to investigate the dilational viscoelasticity (E) in a low-frequency range (from 5 mHz to 0.2 Hz). It was obtained from the surface tension variation due to applying a low amplitude sinusoidal oscillation to the droplet area.

It is well known that there is a correlation between the interfacial properties of the surfactant system and the formation and stabilisation of the corresponding dispersed systems. Therefore, to better understand the changes in foam stability, the dilational elastic modulus was determined for the non-equilibrium conditions during the course of adsorption. In this way, it was possible to monitor the evolution of the viscoelasticity during the ageing of the surface. The experiments were performed applying three frequencies: 5 mHz, 40 mHz and 0.1 Hz. In standard practice, a fixed oscillation was imposed on a fresh droplet during the adsorption re-equilibration of the adsorbed layer.

### 2.7. Confocal Microscopy of Foams

Carl Zeiss LSM780 (Carl Zeiss, Jena, Germany) confocal microscopy was applied to examine the foam structure and the ageing evolution visually. We used microscope glass Petri dishes designed for ellipsometry and confocal microscopy. The foam was generated in the foam column described above. A trace amount of rhodamine was added to the studied mixtures to obtain the fluorescence properties. We checked that the presence of rhodamine had not influenced foamability or foam stability properties. For confocal microscopy evaluation, we took small foam samples from the top of the foam fraction (dry foam).

### 2.8. Size and Charge of Particles

Malvern Zetasizer Nano ZS (Malvern, Worcestershire, UK) with a laser beam at 633 nm with disposable measurement cells (DTS 1065, Malvern) was used for hydrodynamic diameter, polydispersity, and particle charge verification. Zeta potential was determined from the electrophoretic mobility of particles using the Smoluchowski model. Each value was obtained as an average from three consecutive measurements with at least 12 runs. Three experimental processes were performed for each sample, and the average value from all experiments was analysed. We considered differences in refractive index and absorbance at 633 nm. The average error (standard deviation) of zeta potential measurement was about 2 mV maximum.

### 2.9. Bulk Viscosity

The bulk viscosity was measured using a ViscoClock (SI Analytics GmbH, Mainz, Germany) electronic time-measuring unit combined with a Micro-Ubbelohde viscometer (SI Analytics GmbH, Mainz, Germany) with the set of capillaries that allow measuring of the viscosity range between 0.35 and 10,000 mm^2^/s (cSt). The accuracy of the flow time determination was 0.01% with a 95% confidence level. All measurements were performed at 22 ± 1 °C.

### 2.10. Glass Cleaning Methodology

A general cleaning procedure was applied [78] for the glassware: (i) was cleaned using a household detergent and rinsed with hot tap water; (ii) was stored overnight (minimum of 10 h) in the container filled with the solution of the Mucasol universal detergent purchased from Aldrich (high pH); (iii) was rinsed again with hot tap water and cleaned with the mixture of the chromic acid and concentrated sulfuric acid; (iv) was rinsed with twice distilled water and finally with Mili-Q ultrapure water (conductivity 0.05 μS/cm).

### 2.11. General Conditions of Experiments

All experiments were carried out at room temperature of 22 ± 1 °C, using Merck Millipore Direct-Q3 (Merck KGaA, Darmstadt, Germany) ultrapure water (conductivity 0.05 μS/cm, resistivity 18.2 MΩ cm, surface tension 72.4 mN m^−1^ at 22 °C).

## 3. Results

### 3.1. Surface and Foaming Properties of Chitosan, Lauroyl Ethyl Arginate and Acetic Acid Solutions and Mixtures

To properly assess the results of the properties of foams stabilised by silica nanoparticles, firstly we examined the surface activity, dilational elasticity, foamability and foam stability of chitosan, acetic acid and LAE solutions and their mixtures. The key to the analysis of subsequent experiments is evaluating the surface-active and foaming properties for the following solutions: 100 ppm LAE; 100 ppm LAE in the presence of 1% wt. acetic acid; 100 ppm of chitosan in the presence of 1% wt. acetic acid. These analyses’ results are shown mainly in the Supplementary Materials for this paper. Therefore, in the main text of the publication, we will only present discussions of these results with references to figures in Supplementary Materials.

**Figure 1:**
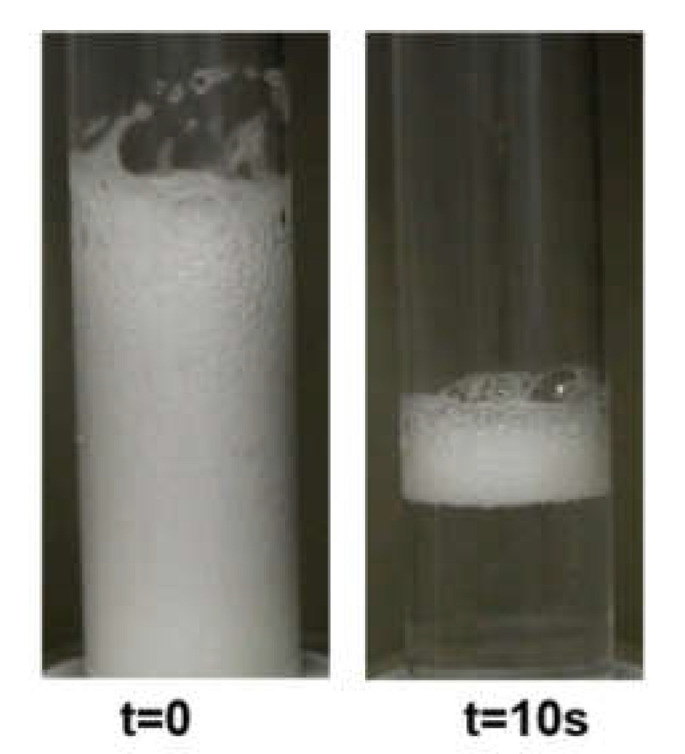
The foam evolution in the chitosan 100 ppm in the presence of 1%wt. acetic acid.

Chitosan, as a hydrophilic polyelectolyte, has practically no surface activity. However, in chitosan 100 ppm solution with the acetic acid presence (1%wt.), we observed a slight decrease in surface tension compared to the values of pure water at the same temperature (see Figure S1 in Supplementary Materials). This is due to the surface-active properties of acetic acid. Furthermore, the studies of the foaming properties of 100 ppm chitosan solution (1%wt. acetic acid) in the foam column show the possibility of producing a relatively high foam fraction. However, generated foam disintegrates rapidly within a few seconds (Figure 1).

Lauroyl ethyl arginate (LAE) is a cationic surfactant. Its 100-ppm solution lowers the surface tension to about 40 mN/m in the time about the 1500 s. The addition of acetic acid does not reduce the surface tension equilibrium values (cf. Figure S2). However, it significantly accelerates the kinetics of adsorption and enables the achievement of values close to equilibrium within the 500 s (Figures S1 and S2). The observed acceleration of the adsorption kinetics of LAE in the presence of acetic acid may be the result of the joint competitive adsorption of LAE cationic surfactant and acetic acid. However, it may also be the result of the action of acetic acid on the cationic surfactant, in which the acid acts as an excess of electrolyte, i.e., it is a source of ionic strength. As a result of this interaction, the double adsorption layer is compressed around the molecules of the ionic surfactant, increasing its surface activity [79,80]. Both processes could also occur simultaneously. It was possible to generate high foams in the 100 ppm LAE solution (both in the presence and absence of 1%wt. acetic acid). However, the developed foams were characterised by a short lifetime of a few dozen seconds. Figure S3 shows the most stable of the described foams obtained with LAE solution in the presence of acetic acid.

A mixture containing all the above-described components, i.e., 100 ppm LAE, 100 ppm chitosan and 1% wt acetic acid, also didn’t show significantly better surface activity (Figures S1 and S2) or foamability (Figure S4) properties than its components. The equilibrium surface tension was only a few percent smaller than in the case of the mixture of LAE with acetic acid. Similarly, the height of the foam portion was ca. 10–15% larger than in the case of the chitosan or LAE solutions. The stability of the foam was also only slightly higher. Although ca. 80% of the foam disappeared within ca. 3 min of its generation, the residue foam fraction was noticeable even after 30 min. This indicates that the ageing process of the foams was prolonged, but it was still a metastable wet foam.

**Figure 2:**
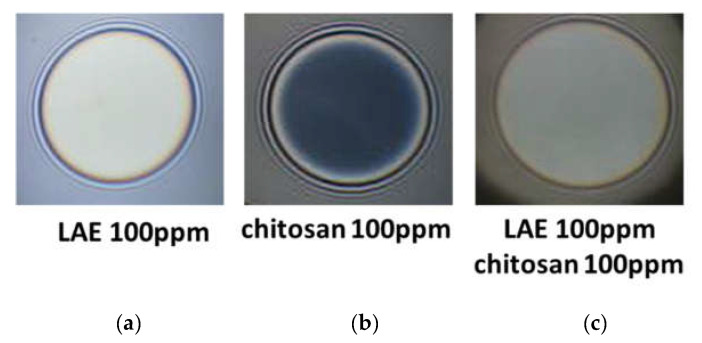
Snapshots of microscopic foam films from aqueous solutions of: (**a**) lauroyl ethyl arginate LAE 100 ppm; (**b**) chitosan 100 ppm in the presence of 1%wt acetic acid; (**c**) the mixture of lauroyl ethyl arginate LAE 100 ppm with chitosan 100 ppm in the presence of 1%wt acetic acid. The snapshots were obtained using Scheludko-Exerowa cell in thin liquid film instrumentation.

**Figure 3:**
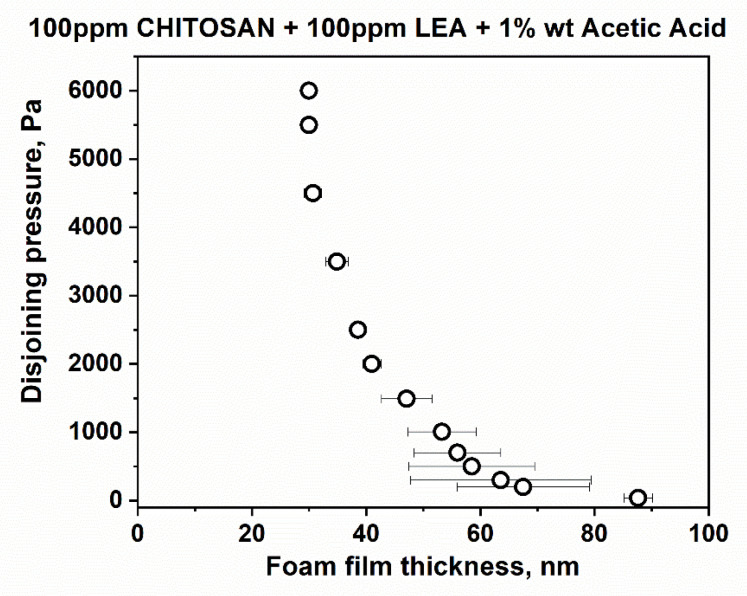
Disjoining pressure vs. film thickness isotherms of microscopic foam films, obtained using porous plate in TLF-PBT.

Figure 2 presents typical snapshots of microscopic foam films from aqueous solutions of lauroyl ethyl arginate 100 pp, chitosan 100 ppm in the presence of 1%wt acetic acid and the mixture of lauroyl ethyl arginate 100 ppm with chitosan 100 ppm in the presence of 1%wt acetic acid. There is a well-outlined difference between single-component and mixed solutions. In the case of the lauroyl ethyl arginate 100 ppm solution (Figure 2a), only unstable thick films are formed. Some of the chitosan-only solutions’ films are stable and have an equilibrium thickness of 52 ± 4 nm (Figure 2b); they rupture at a critical pressure of P_cr_~90 ÷ 105 Pa and h = 29 ÷ 30 nm. Another portion of the films is less stable and ruptures at P_cr_~15 Pa and at equilibrium thickness values. However, the films of LEA and chitosan mixtures are stable with equilibrium thickness values of 84.4 ± 3.5 nm (Figure 2c); critical pressures reach P_cr_~6000 Pa, and the critical film thickness values are h ~ 30 nm (Figure 3).

The microscopic foam film results are in synchrony with the foam tests data described above. The mixed systems definitely stabilise the foam films as components of the foams. However, the foamability and overall foam stability are also related to both the film dimensions, which change during the foam lifetime, and to the consecutive drainage phenomena in the foam channels. If fast drainage is observed, this may quickly destroy almost the whole foam fraction.

### 3.2. Surface and Foaming Properties of Separate Compounds (Chitosan or LAE) in the Presence of Silica Hydrophilic Nanoparticles

We used for the research three types of hydrophilic silica nanoparticles with different sizes: Levasil^®^ 100/45%—30 nm, Levasil^®^ 300/30%—9 nm, and Levasil^®^ 500/15%—6 nm (sizes are given following the manufacturer’s characteristics).

Levasil^®^s are hydrophilic silica particles dispersed in water and presenting a negative surface charge warranting the dispersion stability without further additives. The zeta potential of these particles in water mixtures (no other additives) showed that they have a surface potential ranging from −50 to −60 mV, depending on the type of particles (-54 mV for particles 6 nm; −50 mV for particles 9 nm; −58 mV for particles 30 nm).

In mixtures with 100 ppm chitosan solutions and 100 ppm LAE cationic surfactant, the surface potential of the particles changed to the value: −10.9 mV for 6 nm particles; +56.3 mV for particles 9 nm; +39.4 mV for particles 30 nm.

In foam tests, the effects of chitosan on silica nanoparticles were first investigated. We obtained a high foam in these experiments, but the foam fractions were unstable or metastable. In the case of small particles (Levasil^®^ 300/30%, 6 nm and Levasil^®^ 500/15%, 9 nm), the generated foam disappeared immediately. Slight foam stabilisation was observed only for the largest nanoparticles (Levasil^®^ 100/45%, 30 nm). In experiments with these nanoparticles, more than half the height of the foam fraction disappeared within several or several dozen seconds. The remains of the foam fraction on the surface of the solution (less than 10% of the initial height of the foam) were detected for up to 30 min. Figure S5 presents the best-obtained results describing the foam produced in chitosan 100 ppm/acetic acid 1%wt. mixture with the Levasil^®^ 100/45% nanoparticles. The obtained foams have better stability than the initial solutions of chitosan alone or the LAE cationic surfactant alone (at least in the case of the largest nanoparticles). However, they are not satisfactory from a technological point of view because most of the foam fraction disappeared in less than one minute.

In the case of the LAE solution at 1%wt. acetic acid, the presence of silica particles leads to a relatively stable foam layer. In the case of small particles 6 and 9 nm, the foam disappears in a few minutes (between 1 and a maximum of 5 min).

### 3.3. Zeta Potential of Studied Solutions and Mixtures

The effect of acetic acid, chitosan and LAE on the change of the zeta potential of particles is presented in Table S1 in Supplementary Materials. The presence of LAE in water did not affect the value of zeta potential, while the acetic acid (1%wt.) slightly reduced the negative zeta potential of silica particles. On the contrary, changes in zeta potential prove that chitosan was effectively adsorbed on the surface of particles. The zeta potential was turned into positive values for 30 and 9 nm particles, whereas for 6 nm particles, it remained negative − similar to one in the presence of acetic acid.

In mixtures with 100 ppm chitosan solutions with 100 ppm LAE surfactant, the surface potential of the particles changed to the value: −11 mV for 6 nm particles; +56 mV for particles 9 nm; +39 mV for particles 30 nm, which was similar to chitosan solution without LAE. The results show that, in the case of the smallest particles 6 nm, the amounts of chitosan and acetic acid used are insufficient to completely neutralise the particles’ negative surface charge.

### 3.4. Surface and Foaming Properties of Chitosan/LAE Mixture in the Presence of Silica Nanoparticles

**Figure 4:**
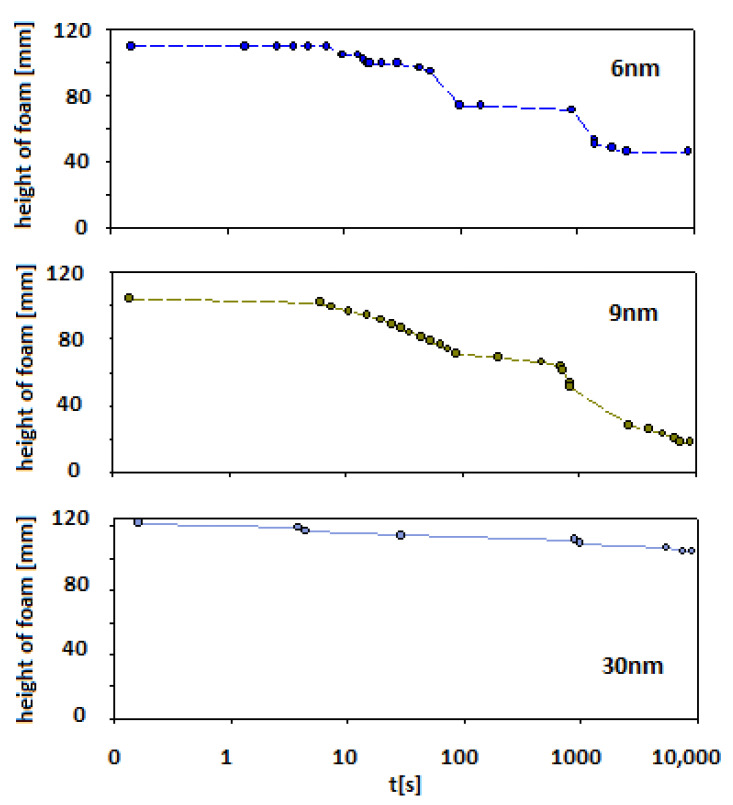
The foam ageing in chitosan—LAE—Levasil^®^ mixtures (depending on the type of Levasil^®^ used).

Figure 4 presents the impact of the three kinds of Levasil^®^ nanoparticles on foam properties composed of the mixture containing chitosan 100 ppm with acetic acid 1%wt. and 100 ppm LAE cationic surfactant.

In the case of smaller particles (Levasil^®^s 500/15% and 300/30%—ca. 6 and 9 nm, respectively) also, stable foams were observed (at least half of the initial height of foam was still observed after 15 min of the experiment). However, after this ageing period, the foam started to disappear to less than 20% of the initial foam column height for 6 nm particles and 40% for 9 nm particles, respectively.

For Levasil^®^ 100/45% (particles size ca. 30 nm), the obtained foam was very stable and existed on almost the initial (t_0_) level even after 3 h. Some traces of the foam fraction were still observed even after three days. Keeping in mind that in each case, the number of added nanoparticles corresponds to the same surface area (90 m^2^), it can be assessed that the stability of the foams varied proportionally with the particle size.

**Figure 5:**
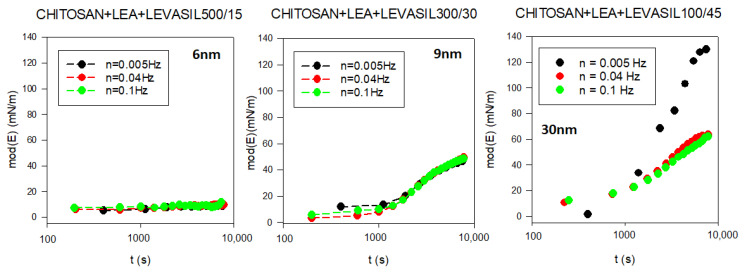
Evolution of the surface dilational elasticity module in chitosan—LAE—Levasil^®^ mixtures (depending on the type of Levasil® used).

To assess the influence of nanoparticles on the interfacial layer properties, we performed surface rheological measurements. As the surface dilational elasticity results were to be directly compared with the lifetime of the foams created based on studied solutions, we applied a non-standard measurement method. A rheological measurement describing adsorption film elasticity is made in the standard way when the equilibrium adsorption coverage is already achieved. In our case, we wanted to investigate changes in elasticity modulus parameters with the adsorption time. Therefore, we conducted a continuous measurement in which the rheological parameters of the adsorption layer were tested several times per minute for up to 3 h of the experiment. The results are presented in Figure 5. As demonstrated, in the dilational surface elasticity moduli case, there is also a relationship to the particle size. The smallest particles of the order of 6 nm (Levasil^®^ 500/15%) practically do not influence surface elasticity. On the other hand, particles with a larger size of 9 and 30 nm increase the elasticity modulus of the layer. The most significant effect was observed for particles with the size of 30 nm (Levasil^®^ 100/45%). These results are consistent with the stability of the foams observed.

In fact, the increase in stability of foams and the values of the elasticity modulus with the size of nanoparticles are coherent with the well-known expression of the transfer energy of a spherical particle from bulk to interfaces used in many experimental works on this topic [81,82]. This relation, for a given wettability of particles, provides a dependence of such energy with the square power of the particle radius, ΔE~R^2^, which means that the attachment is energetically favoured for larger particles, or larger particles irreversibly attach at the interface.

**Figure 6:**
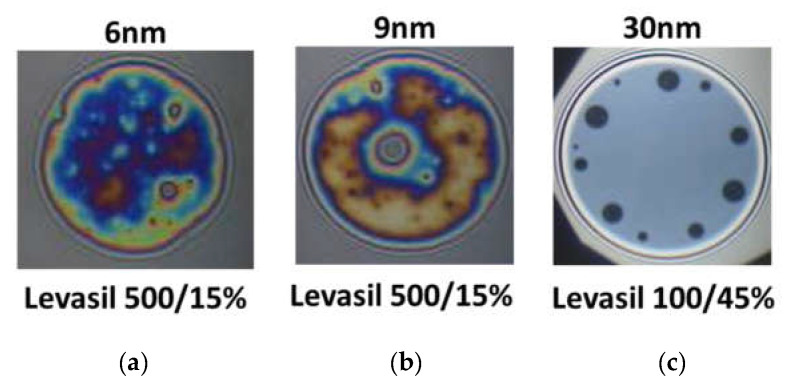
Snapshots of thin liquid foam films prepared from aqueous systems of chitosan 100 ppm, acetic acid 1%wt., lauroyl ethyl arginate LAE 100 ppm mixtures containing various kinds of Levasil^®^. (**a**) 6 nm; (**b**) 9 nm; (**c**) 30 nm. The snapshots were obtained using Scheludko-Exerowa cell in thin liquid film instrumentation.

Figure 6 presents snapshots of microscopic foam films obtained from the mixtures of chitosan 100 ppm, LAE 100 ppm cationic surfactant, and acetic acid 1%wt. with various kinds of Levasil^®^s. Nanoparticles with a size of 6 and 9 nm lead to the formation of rheologically very stable, thick and heterogeneous films in which there was practically no liquid drainage (Figure 6a,b).

On the other hand, in the case of the largest particles with a size of 30 nm, only metastable grey films were obtained, which were ruptured via black film formation (see Figure 6c). These observations’ results contrast with the results of foam stability and surface dilational elasticity studies. However, this result is related to the physicochemical properties of the solution containing fractions of large 30 nm Levasil^®^ particles.

We observed that in the presence of acetic acid, no precipitation in the films was seen. However, these films have a large area of uniform thickness combined with several well-outlined black spots, which are enlarged and merged during the film drainage time (~2 min). This qualitative observation evidences that the large particles in the film bulk are expelled in the film meniscus, and the film ruptures after that. If this hypothesis is correct, in foams the largest particles will gather and/or aggregate in the Plateau channels and plug them, resulting in an enhancement of foam lifetime.

Additional studies on changes in the size of nanoparticles and their aggregates were carried out utilising Dynamic Light Scattering DLS. It proved that each of the studied silica nanoparticles aggregates after mixing with chitosan. The aggregates were hundreds of nanometers in size. In the case of 30 nm particles (Levasil^®^ 100/45%), the dimensions of the observed aggregates exceeded thousands of nanometers. The results confirmed the formation of particle aggregates with dimensions beyond the measurement capabilities of the apparatus (over 1000 nm) for each size of the tested particles in solutions with chitosan and LAE cationic surfactant. The aggregates could fast precipitate and sediment. As a result, a clean solution without particles and a thick layer of precipitated particles can be observed (see Figure S6 in Supplementary Materials).

This hypothesis is also supported by the results of dynamic bulk viscosity measurements of solutions, which prove that the mixture with the 30 nm nanoparticle has more than 5% lower viscosity value than mixtures containing other smaller nanoparticles (6 or 9 nm). The results of bulk viscosity changes in the tested mixtures of silica particles are presented in Table S2 in Supplementary Materials.

The sedimented particles, however, take part in the foaming process because they are transferred to the froth fraction in the flotation process. As a result, the presence of particles improved the stability of the foam (Figure 4).

**Figure 7:**
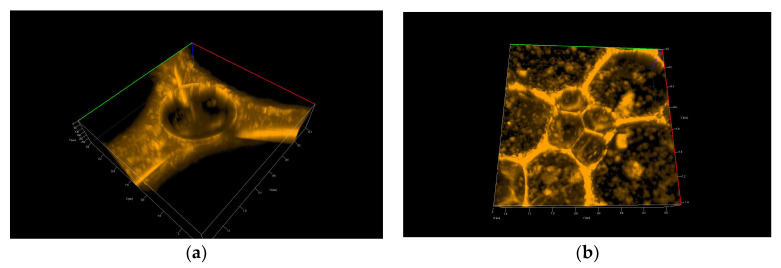
Confocal microscopy photo of 3D foams generated from the chitosan—LAE—Levasil® mixtures: Levasil® 500/15% 6 nm (**a**) and Levasil® 300/30% 9 nm (**b**).

**Figure 8:**
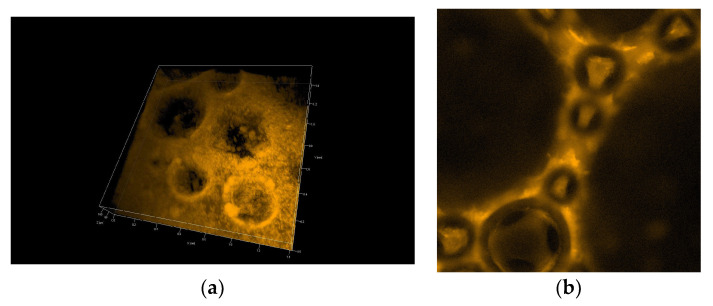
Confocal microscopy photo of the foam generated from the chitosan—LAE—Levasil^®^ 100/45% (30 nm) mixture—3D structure (**a**) and 2D photo (**b**).

Figure 7 and Figure 8 show 3D pictures taken with a confocal microscope of the different froth fractions obtained in a glass column during the foam experiment. A freshly prepared foam fraction from the top of the column was carefully picked up and placed inside the confocal microscope cell. Figure 7 presents 3D images of foams generated based on solutions containing silica particles with 6 nm and 9 nm diameters (Figure 7a—6 nm; Figure 7b—9 nm). Figure 8 shows foams produced in mixtures containing 30 nm particle fractions. (Figure 8a—3D image, Figure 8b—2D). In addition, in Supplementary Materials, four videos show the foam fraction concentration in mixtures containing: 6 nm particles—Videos S1 and S2; 9 nm particles—Video S3 and 30 nm particles—Video S4.

Analysing the photos in Figure 7 and, in particular, the Videos S1–S3 related to them allows for formulating several important qualitative statements summarising the behaviour of small particles in the foam fraction.

Video S1 presents the lamella thin film and Plateau borders in the foams generated in chitosan/LAE mixtures containing 6 nm silica nanoparticles. The confocal microscope did not work here in the three-dimensional scanning mode but showed one unchanged cross-section through the surface layer. We can see how the drainage of nanoparticle-rich liquids reduces the film’s thickness. During the leakage of the liquid, we can observe a sharp change in the structure of the foam in about 15 s, associated with the breakage of one of the adjacent foam cells.

Video S2 shows another place in the thin lamella film generated in mixtures containing 6 nm silica nanoparticles. Here we see the 3D structure of the Plateau borders of the film. In the centre of the Plateau border, the big microbubble is trapped. A thick layer of attached nanoparticles appears on its surface (the film’s beginning), which counteracts the processes of gas diffusion and coalescence (the same situation is presented in Figure 7a).

Video S3 depicts the foam 3D structure obtained from the mixture with 9 nm Levasil^®^ nanoparticles. Most of the space constituting a thin lamellar film is completely filled with nanoparticles (see also Figure 7b). The lack of any evolution of the foam and its cells during the recording allows us to assume that the presence of small nanoparticles (9 nm) leads to the gelation of the liquid in the thin lamellar film layer.

We can conclude that small particles practically do not directly affect the liquid drainage rate from the foam fraction. However, they can indirectly influence the outflow of liquids through micro-bubbles, the interfaces of which can be fully covered by tiny nanoparticles. In this case, these microbubbles behave as solid particles, and their presence blocks the outflow of liquid from the lamella films and Plateau borders. On the other hand, if there are no such microbubbles in the foam, the fluid drains out of the foam film, which becomes thinner up to the minimum limits and then breaks. In addition, small nanoparticles settling in lamellar spaces inhibit coarsening and coalescence, thus extending the foam fraction’s life. As a result, gelation may occur, and the foam fraction can be practically permanently stabilised (as observed in the case of 9 nm particles—Video S3 and Figure 7b).

On the other hand, in the case of large 30 nm nanoparticles, the aggregation of particles leads to the formation of large micro-particles, which practically fill the entire liquid space between the gas bubbles in the foam. The microparticles and microbubbles ultimately effectively block the drainage (see Figure 8 and Video S4).

## 4. Discussion and Summary

This study shows that it is possible to achieve an appropriate hydrophobisation of colloidal silica particles employing trace concentrations of biodegradable compounds, such as biopolymer, polycation chitosan and cationic surfactant lauroyl ethyl arginate.

Cationic surfactant lauroyl ethyl arginate LAE acts as the hydrophobisation agent, while chitosan and partially acetic acid neutralise the particles’ negative surface potential and facilitate their aggregation. As a result, nanoparticles can attach to the gas/solution interface or aggregate into larger systems undergoing spontaneous precipitation (diameter over 1000 nm).

Spontaneous aggregation of particles under the influence of chitosan also reduces their surface area, which makes LAE surfactant more effective in hydrophobising particles. The results from dilational rheology also confirm this hypothesis. The larger the particle size at a given LAE concentration, the more favoured the formation of a viscoelastic layer at the water–gas interface.

The presence of small particles (6–10 nm) in the solution affects its viscosity, slows down liquid drainage from the foam lamellar thin films and blocks coalescence and gas diffusion.

Hydrophobised small nanoparticles (6–10 nm) may also be able to adsorb the microbubbles formed during the foaming process. Such bubbles, with their interfacial surface practically fully covered by nanoparticles, do not undergo the processes of coalescence and gas diffusion, remaining the same shape and size practically regardless of the lifetime of the foam.

The obtained results are consistent with those processes previously observed by various authors of increasing the viscosity of the liquid containing nanoparticles [25], gelation of the foam layer related to the presence of a large number of small nanoparticles [26] and hydrophobisation of particles in solutions by appropriate surfactants [40,41,42,43,44,45,46,47].

During the foaming process, large micrometric particles and aggregates (previously lost from the solution by sedimentation) are brought into the foam layer in the froth flotation process. Large micrometric gas bubbles and microparticles in the lamellar film layer and Plateau borders lead to practically complete inhibition of liquid drainage.

The synergistic interaction of chitosan and lauroyl ethyl arginate with silica nanoparticles allows for obtaining a stable and mechanically resistant foam layer at LAE surfactant concentrations many times (more than ten times) lower than its critical micelle concentration (LAE CMC ca. 1 g/L [46,47,48,49,50,51,52,53,54,55,56,57,58,59,60,61]).

The developed method can be used for hydrophobisation and removal of colloidal impurities from wastewater solutions. In addition, other particles can be used to generate safe, stable foam fractions for use in the cosmetics industry or medical applications.

## Figures and Tables

**Table 1:** The Levasil^®^ volumes used for chitosan/Levasil^®^ mixture formulations.

Levasil^®^	Surface Area 90 m^2^ per 100 cm^3^ of Mixture
100/45%	2.0 cm^3^
300/30%	1.0 cm^3^
500/15%	1.2 cm^3^

## Data Availability

The data presented in this study are freely available from the authors upon a reasonable request.

## Supplementary Materials

The following supporting information can be downloaded at: https://www.mdpi.com/article/10.3390/polym14194076/s1, Figure S1: The dynamic surface tension of chitosan and lauroyl ethyl arginate in water and in the presence of 1%wt. chitosan; Figure S2: Surface tension variation in chitosan 100 ppm–LEA 100 ppm—acetic acid 1%wt. mixture; Figure S3: The foam evolution in the LAE 100 ppm in the presence of 1%wt. acetic acid; Figure S4: Foaming properties in the mixture of chitosan 100 ppm, LEA 100 ppm and acetic acid 1%wt.; Figure S5: The foam from chitosan/acetic acid mixture with the Levasil^®^ 100/45% (30 nm) nanoparticles; Table S1: The zeta potential change of silica particles under the influence of acetic acid, chitosan and lauroyl ethyl arginate LAE; Table S2: Bulk viscosities of silica particles under the influence of acetic acid, chitosan and lauroyl ethyl arginate LAE; Video S1 Foam evolution—foam fraction formed from 6 nm Levasil^®^ particles; Video S2: Foam evolution—foam fraction formed from 9 nm Levasil^®^ particles; Video S3: Foam evolution—foam fraction formed from 6 nm Levasil^®^ particles—effect of microbubbles; Video S4: Foam evolution—foam fraction formed from 30 nm Levasil^®^ particles.
