# Supplementary material for: Hydrophobisation of Silica Nanoparticles Using Lauroyl Ethyl Arginate and Chitosan Mixtures to Induce the Foaming Process
Source: Polymers (Basel). 2022 Sep 28;14(19):4076. doi: 10.3390/polym14194076 (PMC9573722; doi:10.3390/polym14194076)
Supplement: Supplementary file 1 [file polymers-14-04076-s001.zip › Supplementary Materials proofreading version.pdf]

Supplementary Materials

Hydrophobisation of silica nanoparticles using lauroyl ethyl arginate and chitosan mixtures to induce the foaming process

Marcel Krzan[1*], Ewelina Jarek[1], Hristina Petkova[2], Eva Santini[3], Lilianna Szyk-Warszynska[1], Francesca Ravera[3], Libero Liggieri[3], Elena Mileva[2] and Piotr Warszynski[1]

[1] Jerzy Haber Institute of Catalysis and Surface Chemistry, Polish Academy of Sciences, ul. Niezapo-minajek, 8, 30-239 Krakow, Poland
[2] Institute of Physical Chemistry, Bulgarian Academy of Sciences, Acad. G. Bonchev Str., bl.11, 1113 Sofia, Bulgaria
[3] Institute of Condensed Matter Chemistry and Technologies for Energy, Consiglio Nazionale delle Ricerche, Via Marini 6, 16149 Genoa, Italy
*** Correspondence: marcel.krzan@ikifp.edu.pl**

List of materials :

1. Figure S1: The dynamic surface tension of chitosan and lauroyl ethyl arginate in water and in the presence of 1%wt. chitosan;
2. Figure S2: Surface tension variation in chitosan 100 ppm–LEA 100 ppm—acetic acid 1%wt. mixture;
3. Figure S3: The foam evolution in the LAE 100 ppm in the presence of 1%wt. acetic acid;
4. Figure S4: Foaming properties in the mixture of chitosan 100 ppm, LEA 100 ppm and acetic acid 1%wt.;
5. Figure S5: The foam from chitosan/acetic acid mixture with the Levasil® 100/45% (30 nm) nanoparticles;
6. Table S1: The zeta potential change of silica particles under the influence of acetic acid, chitosan and lauroyl ethyl arginate LAE;
7. Table S2: Bulk viscosities of silica particles under the influence of acetic acid, chitosan and lauroyl ethyl arginate LAE;
8. Video S1 Foam evolution—foam fraction formed from 6 nm Levasil® particles;
9. Video S2: Foam evolution—foam fraction formed from 9 nm Levasil® particles;
10. Video S3: Foam evolution—foam fraction formed from 6 nm Levasil® particles—effect of microbubbles;
11. Video S4: Foam evolution—foam fraction formed from 30 nm Levasil® particles.

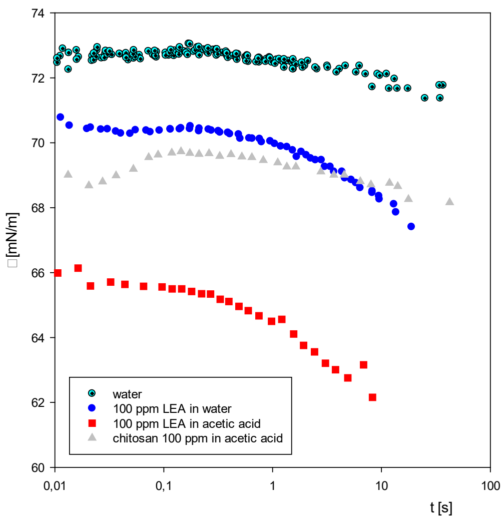

Figure S1 The dynamic surface tension of chitosan and lauroyl ethyl arginate in water and in the presence of 1%wt. chitosan.

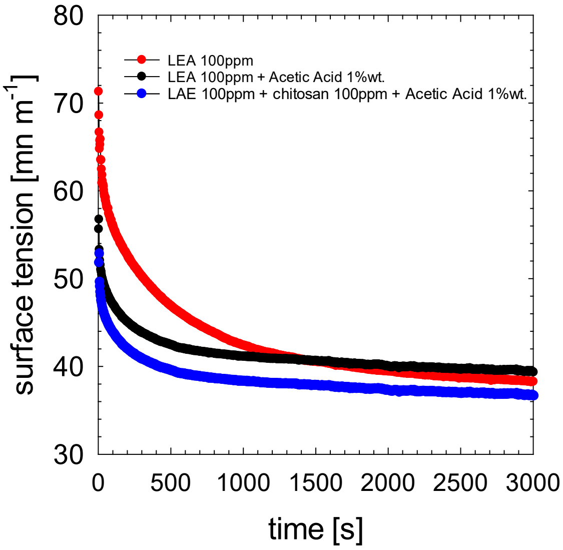

Figure S2. Surface tension variation in chitosan 100 ppm– LEA 100ppm - acetic acid 1%wt. mixture.

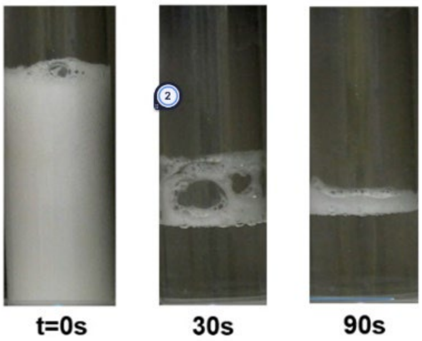

Figure S3 The foam evolution in the LAE 100ppm in the presence of 1%wt. acetic acid.

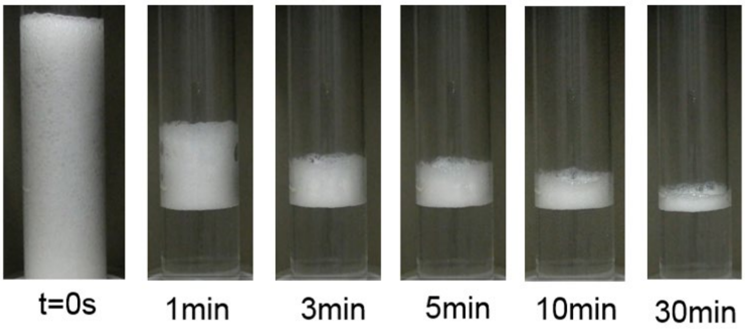

Figure S4 Foaming properties in the mixture of chitosan 100ppm, LEA 100ppm and acetic acid 1%wt.

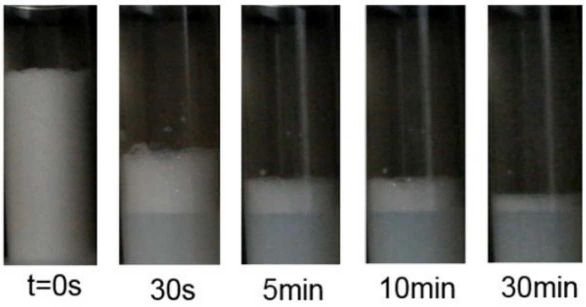

Figure S5 The foam from chitosan/acetic acid mixture with the Levasil® 100/45% (30nm) nanoparticles.

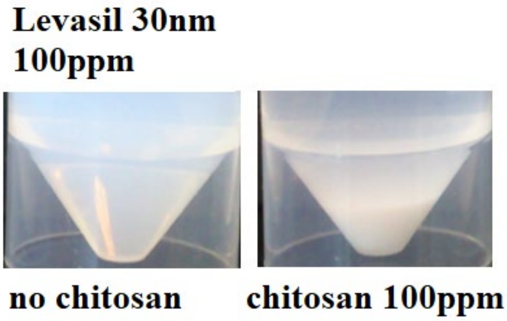

Figure S6 Sedimentation of the precipitated particles of Levasil® 100/45% in the presence of chitosan 100ppm

Table S1. The zeta potential change of silica particles under the influence of acetic acid, chitosan and lauroyl ethyl arginate LAE.

Levasil® Silica	Water	Acetic acid 1%wt.	Chitosan / Acetic Acid 1%wt.	LAE in water	LAE / Acetic Acid 1%wt.	LAE / Chitosan / Acetic Acid 1%wt.
30 nm	-54mV	-17mV	39mV	-54mV	-14mV	39mV
9nm	-50mV	-22mV	52mV	-52mV	-29mV	56mV
6nm	-57mV	-19mV	-19mV	-58mV	-29mV	-11mV

Table S2. Bulk viscosities of silica particles under the influence of acetic acid, chitosan and lauroyl ethyl arginate LAE.

Levasil® Silica nanoparticles	Water (cSt)	Acetic acid 1%wt. (cSt)	Chitosan / Acetic Acid 1%wt. (cSt)	LAE / Chitosan / Acetic Acid 1%wt. (cSt)
30 nm	0.99	1.03	1.18	1.17
9nm	0.98	1.02	1.30	1.22
6nm	1.01	1.04	1.30	1.22
